# The many faces of Pluripotency: in vitro adaptations of a continuum of in vivo states

**DOI:** 10.1186/s12861-017-0150-4

**Published:** 2017-06-13

**Authors:** Sophie Morgani, Jennifer Nichols, Anna-Katerina Hadjantonakis

**Affiliations:** 1 Developmental Biology Program, Sloan Kettering Institute, Memorial Sloan Kettering Cancer Center, New York, NY 10065 USA; 2 Wellcome Trust-Medical Research Council Centre for Stem Cell Research, University of Cambridge, Tennis Court Road, Cambridge, CB2 1QR UK

**Keywords:** Pluripotency, Naïve, Intermediate, Formative, Primed, Embryonic stem cells, Epiblast-like cells, Epiblast stem cells, Ground state, Chimaera

## Abstract

Pluripotency defines the propensity of a cell to differentiate into, and generate, all somatic, as well as germ cells. The epiblast of the early mammalian embryo is the founder population of all germ layer derivatives and thus represents the bona fide in vivo pluripotent cell population. The so-called pluripotent state spans several days of development and is lost during gastrulation as epiblast cells make fate decisions towards a mesoderm, endoderm or ectoderm identity. It is now widely recognized that the features of the pluripotent population evolve as development proceeds from the pre- to post-implantation period, marked by distinct transcriptional and epigenetic signatures. During this period of time epiblast cells mature through a continuum of pluripotent states with unique properties. Aspects of this pluripotent continuum can be captured in vitro in the form of stable pluripotent stem cell types. In this review we discuss the continuum of pluripotency existing within the mammalian embryo, using the mouse as a model, and the cognate stem cell types that can be derived and propagated in vitro. Furthermore, we speculate on embryonic stage-specific characteristics that could be utilized to identify novel, developmentally relevant, pluripotent states.

## Background

**Fig. 1:**
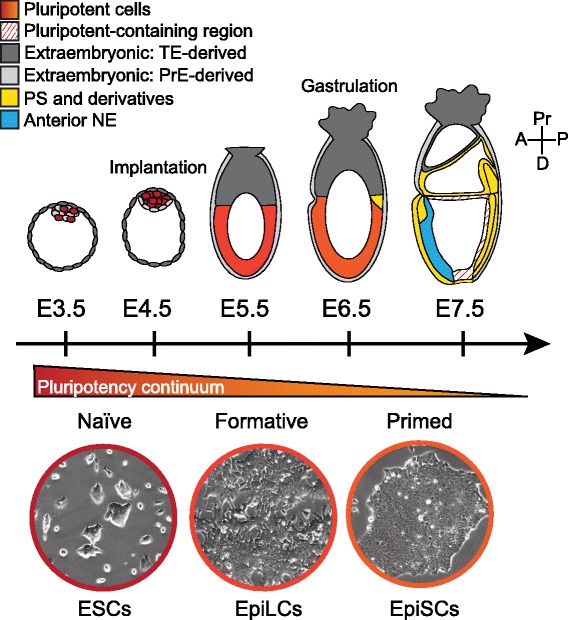
A schematic diagram depicting the relationship between *in vitro* and *in vivo* pluripotent state progression. The diagram depicts the location of pluripotent cells (*red*) within the developing mouse embryo from embryonic day (E) 3.5 to E7.5. Prior to E3.5, cells of the pre-implantation embryo are ‘totipotent’, capable of generating both embryonic and extraembryonic cell types. At E3.5, cells in the ICM of the blastocyst are a heterogeneous mix of epiblast (Epi) and primitive endoderm (PrE) precursors. Epi cells are pluripotent and will generate all cells of the embryo-proper, including the germ cells, proper while PrE cells will generate extraembryonic cell types such as the yolk sac. The outer trophectoderm (TE) cells will generate extraembryonic cell types including the fetal portion of the placenta. One day later, at E4.5, the Epi and PrE cells are specified and become physically segregated into two distinct layers and the embryo implants into the uterus. At early post-implantation stages (E5.5) the Epi is in an entirely undifferentiated pluripotent state. At E6.5, cells within the proximal posterior of the embryo are exposed to differentiation-promoting signals from both embryonic and extraembryonic lineages that stimulate the onset of gastrulation and differentiation of cells as they enter the primitive streak (PS) region (*yellow*). By E7.5, the PS has extended distally and PS derivatives including extraembryonic mesoderm, embryonic mesoderm and definitive endoderm are being generated. The anterior Epi has also started to differentiate into anterior neurectoderm (NE). Pluripotency is lost at approximately E8.0. Pluripotent stem cell lines can be maintained in vitro and appear to resemble various embryonic stages of pluripotency. While embryonic stem cells (ESCs) can be derived from embryos from E3.5 and E7.5 and epiblast stem cells (EpiSCs) can be derived from embryos between E3.5 and E8.0, ESCs resemble the naïve pluripotent state (*blue*) existing in the early pre-implantation embryo while EpiSCs resemble primed pluripotent cells (*green*) of the late post-implantation Epi during gastrulation. Intermediate or formative states of pluripotency (*orange*), between the naïve and primed states likely exist in the embryo. While a number of potential states have been isolated, Epi-like cells (EpiLCs), generated from ESCs in vitro, have been most clearly defined in relation to the embryo and are more transcriptionally similar to E5.75 Epi than EpiSCs are. Representative brightfield images of ESC, EpiLC and EpiSC cultures are shown. Extraembryonic lineages are depicted in *gray*; *dark gray* lineages are TE-derived and *light gray* lineages PrE-derived. A = anterior, P = posterior, Pr = proximal, D = distal

Pluripotency is the potential of a single cell to generate all somatic lineages of the adult organism, comprising mesoderm, endoderm and ectoderm derivatives, as well as the germ cells. During early mammalian development, cells within the epiblast (Epi) of the embryo are pluripotent and go on to form the embryo-proper. As development progresses, a combination of Fibroblast Growth Factor (FGF), Bone Morphogenetic Protein (BMP), Wnt and Nodal signaling triggers the loss of pluripotency by driving differentiation of the Epi into specialized, developmentally-restricted fates [1]. In the mouse, pluripotent cells are present from embryonic day (E) 3.5 to 8.0, representing approximately one quarter of the gestation period (Fig. 1). During this time, the pluripotent population evolves, characterized by changes in gene expression, epigenetic profile and functional properties. While distinct “naïve” and “primed” pluripotent states have been described, that correspond to the pre and post-implantation Epi respectively [2], the progressive nature of development means that a broad continuum of pluripotency likely exists within the developing embryo (Table 1). To define additional intermediate states, a high-resolution gene expression map of these embryonic stages is required, a task made difficult by the rapid advancement of in vivo development and limited availability of material.

**Table 1:** Overview of pluripotent states and defining characteristics. Although naïve and primed states of pluripotency have been well characterized, there is no clear consensus of the expected characteristics of their intermediate pluripotent states. This table highlights a number of defining characteristics of naïve and primed pluripotent states, and stipulates on the characteristics that intermediate states might encompass. Although a spectrum of intermediate states may exist, here we hypothetically distinguish between two potential intermediate states, ‘Intermediate 1’, the epiblast immediately after implantation and ‘Intermediate 2’ the epiblast at the onset of gastrulation

Pluripotent state	Corresponding embryonic stage	Gene expression	Epigenetic profile	Functional potential
Naïve - ESCs	E3.5–4.5	*Oct4*, *Sox2*, *Nanog*, *Klf4*, *Stella*, *Rex1*, *Gbx2*, *Tbx3*, *Pecam*, SSEA-1, Alkaline phosphatase	X reactivation. DE-controlled Oct4 expression.	Pre-imp. Chimaeras. Poor PGCLC generation.
Primed - EpiSCs	E7.25–8.0	*Oct4*, *Sox2*, *Nanog*, SSEA-1, *Fgf5*, *Oct6*, *Otx2*, *Brachyury*, *FoxA2*, *Sox17*, *Gata4*, *Gata6*	X inactivation. PE-controlled Oct4 expression.	Post-imp. Chimaeras. Poor PGCLC generation.
Intermediate 1	E5.0–6.25	*Oct4*, *Sox2*, low *Nanog*, SSEA-1, *Fgf5*, *Oct6*, *Otx2* (no PS or lineage markers)	X reactivation, Equal Oct4 regulation by DE and PE?	Pre and post-imp. Chimaeras. Efficient PGCLC generation.
Intermediate 2	E6.25–7.25	Early PS/mesoderm markers e.g. *Nanog*, *Brachyury*. No endoderm markers.	X inactivation.	Post-imp. Chimaeras. Reduced PGCLC generation.

*ESCs* embryonic stem cells, *EpiSCs* epiblast stem cells, *E* embryonic day, *DE* distal enhancer, *PE* proximal enhancer, *pre-imp.* pre-implantation, *post-imp.* post-implantation, *PGCLC* primordial germ cell-like cell, *PS* primitive streak

It is, however, possible to study pluripotency in a more stable state in vitro using cell lines derived from the embryo [3–6]. These cell lines offer a tool to study early development, as well as a reservoir of unspecified cells with significant therapeutic potential. Pluripotent stem cell (PSC) lines can self-renew indefinitely while maintaining the capacity to differentiate into all cell types in vitro [7–9] and in vivo [4, 10–12]. Many iterations of pluripotency can be propagated in culture depending on the conditions utilized (Table 2). It is currently unclear whether these represent distinct points on a pluripotency spectrum, which also arise in vivo during normal development, or if they are merely culture artifacts. Here, we discuss our current knowledge of PSC states, focusing on the mouse model, in which most research has been carried out.

**Table 2:** Details of mouse pluripotent stem cell culture conditions. This table highlights that there are many variations on culture conditions for maintaining cells in distinct pluripotent states, including subtle differences in basal medium and cytokine concentrations that are often not emphasized. All culture medium formulations contain amino acids and 2-mercaptoethanol as standard. Pluripotency markers include *Oct4*, *Sox2*. Naïve pluripotency markers include *Nanog*, *Rex1*, *Stella*, *Klf4*, *Esrrb*. Primed pluripotency markers include *Fgf5*. Lineage markers include *Brachyury*, *FoxA2*, *Eomes*, *Gata6*, *Gata4*, *Sox17*. ‘Embryo-derived’ refers to cell lines that are directly derived from embryos, whereas ‘culture-derived’ refers to cell lines that are interconverted from different states e.g. changing the culture conditions of ESCs or EpiSCs

Cell type	Culture condition	Added factors	Basal medium	Embryo-derived	Culture- derived	Gene expression	X status	Functional tests	Ref.
Embryonic stem cells (ESCs)	Feeders/ serum	-	MEFs/ DMEM/ 20% serum	✓		Little transcriptional data	✓	IVD, teratomas, pre-imp. Chimaeras, GLT	[4, 12]
	Serum/LIF^‡^	1000 U/ml LIF	GMEM or DMEM/ 10% serum	✓		Naïve markers. Heterogeneous lineage markers.	✓	IVD, teratomas, pre-imp. Chimaeras, GLT.	[27, 28]
	BMP/LIF	10 ng/ml BMP4 OR 200 ng/ml GDF6 + 1000 U/ml LIF	N2B27	✓	✓	Little transcriptional data. Homogeneous *Oct4*		Pre-imp. Chimaeras, GLT.	[29]
	2i*	1 μm PD032/ 3 μM CHIR/ (+/− LIF)	N2B27	✓	✓	Homogeneous naïve markers. Reduced lineage markers (rel. to SL).	✓	IVD, pre-imp. Chimaeras, GLT.	[91, 97]
	KOSR/LIF	1000 U/ml LIF	Knockout DMEM/ 20% KOSR	✓	✓	Naïve and PrE markers	**?**	IVD, pre-imp. Chimaeras, GLT.	[46, 101]
Epiblast stem cells (EpiSCs)	F/A^‡^	5-12 ng/ml FGF2 + 10-20 ng/ml Activin	MEFs, Knockout DMEM/ 20% KOSR or “CDM” [207]	✓	✓	Primed markers. Heterogeneous lineage markers.	✗	IVD, teratoma formation, rare pre-imp. Chimaeras, post-imp. Chimaeras.	[3, 17]
	F4-EpiSC	25 ng/ml FGF4	DMEM/ 20% serum	✓		Homogeneous *Oct4*. Primed and lineage markers.	✗	Teratomas, pre-imp. Chimaeras.	[160]
	F/A/XAV* (WiEpiSC)	5 ng/ml FGF2/ 10 ng/ml Activin/ 10 μM XAV	DMEM/20% KOSR	✓	✓	Homogeneous pluri and primed markers. Reduced lineage markers (relative to FA).	**?**	Post-imp. Chimaeras –bias towards extraembryonic mesoderm.	[156]
	F/A/IWP2* (WiEpiSC)	12 ng/ml FGF2, 20 ng/ml Activin, 2 μM IWP2	Derivation in DMEM/ 18% KOSR/ 2% serum ➔ N2B27 at P2		✓	Pluri markers. Reduced lineage markers (rel. to FA).	**?**	Enhanced ESC reversion, pre-imp. Chimaeras.	[127]
	XAV/CHIR	3 μM CHIR/ 2 μM XAV	GMEM/ 10% serum	✓	✓	Reduced naïve markers (rel. to ESCs). Primed markers.	✗	Teratomas formation.	[20]
Potential intermediate states	EpiLCs	12 ng/ml FGF2, 20 ng/ml Activin	N2B27/ 1% KOSR		✓	Reduced pluri/naïve markers. Increased primed markers (rel. to ESCs). Reduced lineage markers (rel. to EpiSCs)	✗	Efficient PGCLC generation.	[145]
	Transient Epi state	10-12 ng/ml FGF2	N2B27 (+/− 1% KOSR)		✓	Decreased *Rex1,* Increased *Fgf5*.	**?**	In vitro NMPs.	[154, 155]
	INTPSC	12 ng/ml FGF2, 10 ng/ml Activin, 3 μM CHIR	N2B27/ 1% KOSR		✓	Intermediate expression of pluri, naïve, primed and lineage markers.	✓	Teratoma formation, pre-imp. Chimaeras.	[155]
	FAB-SCs	1 ng/ml FGF2, 50 ng/ml Activin, 0.5 μM BIO, 100 ng/ml LIF blocking antibody	MEFS/ DMEM/ 15% KOSR	✓		Pluri markers. Naïve miRNAs. Reduced naïve/germ cell markers (rel. to ESCs). Little/no primed expression.	**?**	Not functionally pluripotent	[161]
	IESCS	12 ng/ml Activin	GMEM/ 10% serum		✓	Intermediate expression of pluri, naïve, primed and lineage markers.	**?**	IVD, teratoma formation – incorporate into pre-imp. Embryos but negative impact on development.	[162]
	EPL	+/− 1000 U/ml LIF	50% MEDII conditioned medium/ 50% DMEM/ 10% serum		✓	Pluri markers. Reduced naïve and increased primed markers (rel. to ESCs).	**?**	Not functionally pluripotent	

X status refers to the activation status of the X chromosome, ✗ = inactive X chromosome, ✓ = active X chromosome. *DR* downregulated, *UR* upregulated, *Epi* epiblast, *PrE* primitive endoderm, *IVD* in vitro differentiation, *pre-imp.* pre-implantation, *post-imp.* post-implantation, *GLT* germline transmission, *pluri* pluripotency genes. ^‡^ = standard culture condition, * = “ground state” of self-renewal, rel. to = relative to

## Embryonic stem cells

### (i) derivation and culture conditions

Mouse embryonic stem cells (ESCs) were the first PSC lines to be derived from developing embryos. ESCs are routinely derived from and resemble the naïve Epi of E3.5–4.5 pre-implantation embryos [4, 12–14]. While ESCs can also be derived from embryos as early as E0.5 [6, 13, 15], these embryos develop ex vivo to a late blastocyst stage before ESCs emerge [13]. Conversely, attempts to derive PSC lines from later stage embryos using naïve ESC culture conditions have been largely unsuccessful, consistent with the distinct nature of the pre and post-implantation Epi. Intriguingly, while ESC lines cannot be established from whole explants of post-implantation Epi [3, 16, 17], they can be derived from dissociated E7.5 Epi [18, 19] implying that a refractory niche may be present within the intact tissue. These cells, referred to as reprogrammed Epi ESC-like cells (rESCs), undergo transcriptional and epigenetic changes consistent with reprogramming to an earlier developmental state during the derivation procedure [18]. In vitro equivalents of the post-implantation Epi (epiblast stem cells, see below) can also occasionally revert to an ESC state [18–23], a process enhanced by genetic manipulation [16, 24–26]. Pluripotent cells therefore maintain a degree of plasticity and can deviate from, or even reverse, the normal developmental trajectory if a permissive environment is provided.

**Fig. 2:**
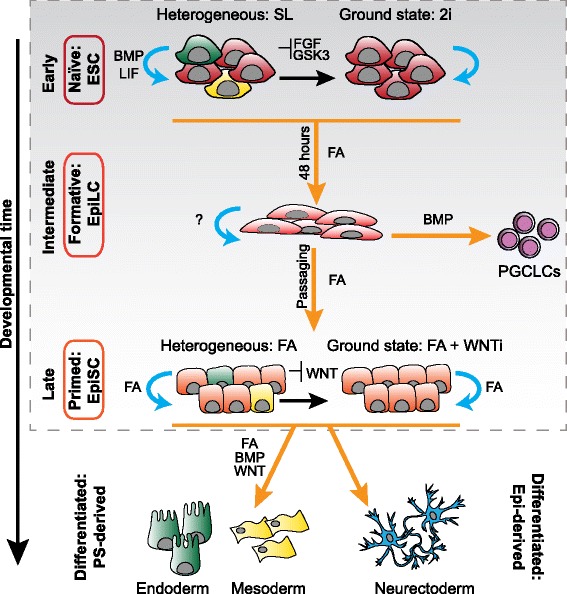
The role of signaling pathways in self-renewal and differentiation of in vitro pluripotent stem cell populations. Embryonic stem cells (ESCs) represent a naïve state of pluripotency similar to the pre-implantation epiblast (Epi). ESCs are routinely maintained in a self-renewing state in serum (a source of BMP) and LIF (SL). Under these conditions, ESC cultures are heterogeneous and contain subpopulations of lineage-primed cells (*yellow* and *green cells*) i.e. cells that coexpress germ layer markers alongside pluripotency markers, and are biased in differentiation towards particular lineages. A more homogeneous ESC state can be generated by blocking FGF signaling using a MEK inhibitor (PD0325901), and activating Wnt signaling using a GSK3 inhibitor (CHIR99021), a condition known as ‘2i’. In these conditions, self-renewal occurs in the absence of external signals, although cell propagation is enhanced in the presence of Wnt pathway activity through CHIR99021. This state is referred to as the naïve or “ground state” of pluripotency. ESCs can be pushed further along the differentiation trajectory by culturing in the presence of FGF and Activin (FA) for 48 hours to generate a cell state referred to as Epi-like cells (EpiLCs). This is a transient cell state, and it is unknown whether self-renewing EpiLCs can be captured by the addition of other factors. It is also not known whether EpiLCs are a homogeneous population of cells. Upon further differentiation in FA over multiple passages, cells resemble a primed state of pluripotency akin to the later post-implantation Epi, referred to as epiblast stem cells (EpiSCs). While FA promotes differentiation of ESCs and EpiLCs, it promotes EpiSC self-renewal. EpiSCs can be derived from ESCs in culture or directly from embryos with FA. When grown in FA, EpiSCs, like ESCs in SL, are heterogeneous and contain lineage-primed populations. While activation of Wnt signaling promotes a naïve ground state of self-renewal, inhibition of Wnt signaling promotes a more homogeneous primed ground state of self-renewal. Therefore cells in naïve and primed pluripotent states respond to signaling factors with opposite outcomes, Wnt and BMP promote self-renewal of the naïve state but differentiation of the primed state of pluripotency and conversely FA promote differentiation of the naïve state but self-renewal of the primed state of pluripotency. Addition of BMP4 and WNT3A in combination with FA stimulates further differentiation of EpiSCs into PS-derived mesoderm and endoderm, while in the absence of FA, BMP or Wnt EpiSCs differentiate to neurectoderm [127]. EpiLCs are the only pluripotent state that has been shown to efficiently generate primordial germ cell-like cells (PGCLCs). Presumably, ESCs have not yet acquired this capacity, while EpiSCs have lost it. Cells within the *dashed box* are within the pluripotency spectrum while cells outside have differentiated. *Blue arrows* indicate self-renewal. *Orange arrows* denote the direction of differentiation along the developmental trajectory

ESCs were first derived in poorly defined serum containing medium and were maintained on a bed of mitotically inactivated fibroblasts (so-called ‘feeder cells’) [4, 12]. The critical factors provided by each of these components are now known to be activators of the leukemia inhibitory factor (LIF) [27, 28] and BMP [29] pathways respectively (Fig. 2, Table 2). While ESCs can be maintained in defined conditions with LIF and BMP4 [29–31] (or other interleukin-6 family members [32–36]), serum and LIF (SL) is favored as an economical alternative with enhanced plating efficiency [29]. LIF and BMP stimulate the expression of pluripotency-associated genes in ESC cultures [29, 31, 37], and BMP may additionally inhibit differentiation-inducing Mitogen-activated protein kinase/Extracellular signal-regulated kinase (MAPK/ERK) signals [38, 39] (Fig. 2). BMP also maintains Epi pluripotency in vivo by preventing premature neural specification [40] and, while LIF is not required for normal pre-implantation development [41–45], it maintains self-renewal during diapause [43]. Therefore, ESCs retain the key signaling properties of their embryonic cell of origin and may be similar to the Epi of diapause embryos.

### (ii) transcriptional and epigenetic profiles

**Fig. 3:**
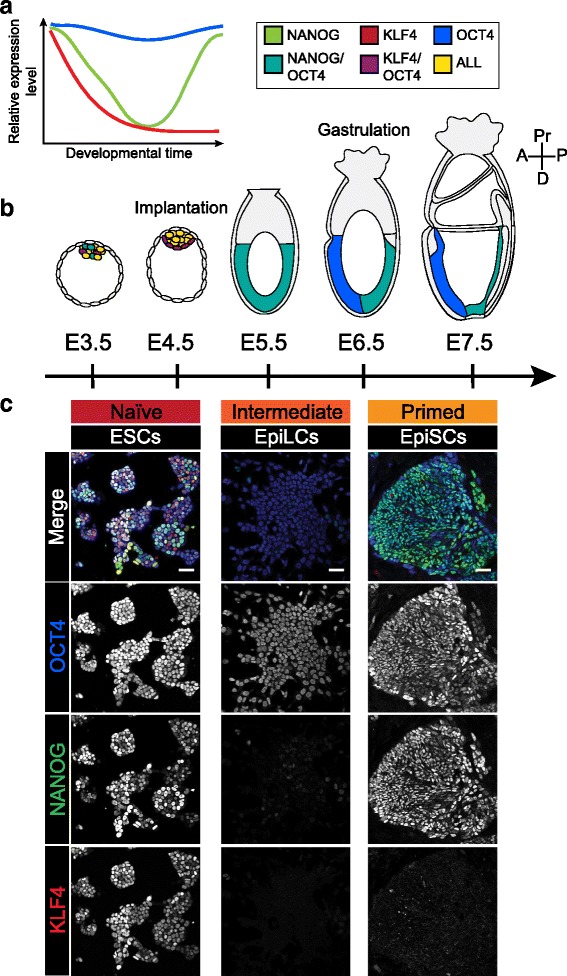
Different pluripotent states have distinct expression profiles. **a**. Schematic diagram illustrating the change in relative protein expression levels of the pluripotency-associated genes, NANOG, KLF4 and OCT4 during the transition from a naïve to a primed state of pluripotency. KLF4 is lost as cells exit the naïve state of pluripotency, NANOG is transiently downregulated and OCT4 is maintained at similar levels throughout this period. **b.** Schematic diagram showing the expression domains of NANOG, KLF4 and OCT4 from embryonic day (E) 3.5 to 7.5 of development. NANOG, KLF4 and OCT4 are all expressed within the ICM of the early blastocyst. While OCT4 is relatively homogeneous, KLF4 and NANOG are both heterogeneously expressed. At E4.5, the epiblast (Epi) homogeneously expresses all 3 of these markers, while the primitive endoderm expresses low levels of OCT4 and KLF4 but not NANOG. At early implantation (E5.5), KLF4 expression is lost and OCT4 and NANOG are coexpressed throughout the Epi. By E6.5–7.5, OCT4 continues to be expressed throughout the Epi while NANOG is restricted to the posterior Epi. **c.** Representative confocal optical sections of ESC, EpiLC and EpiSC cultures. All cell lines were derived from the 129/Ola E14 parental ESC line. ESCs were maintained in serum and LIF and expressed OCT4, NANOG and KLF4 heterogeneously. EpiLCs expressed OCT4, but downregulated NANOG, and lost KLF4 expression. EpiSCs (derived by culture of E14 ESCs in FGF and Activin for >20 passages) expressed high levels of NANOG and OCT4, but no KLF4

Although ESCs can be derived from multiple developmental stages, they retain no clear ‘memory’ of their developmental origin and converge at a transcriptional and epigenetic state similar to the Epi of the E3.5–4.5 blastocyst [13, 46]. ESCs exhibit an open chromatin structure and high levels of global transcriptional activity, similar to the pre-implantation embryo, that become more restricted as differentiation proceeds [47–50]. This active chromatin state is characterized by large regions of DNA hypomethylation, histone acetylation and H2K4me3 [51, 52] and is attributed in part to factors recruited to the citrullination modification on histone H1 [53, 54]. Furthermore, female ESC lines exhibit X chromosome inactivation, an epigenetic hallmark of the naïve pluripotent state present at this time in vivo [55], although the level of X chromosome methylation varies between individual cells [56]. ESCs also express a cohort of transcription factors characteristic of the pre-implantation Epi including *Oct4* (*Pou5f1*), *Sox2*, *Nanog*, *Klf4, Stella (Dppa3*) and *Rex1* (*Zfp42*) [57, 58] (Fig. 3). Furthermore, as in the blastocyst, *Oct4* expression is regulated by its distal enhancer element [59]. Some of the key targets of this transcription factor network include families of micro RNAs (miRNAs) that regulate cell cycle progression in the self-renewing state [60–62]. These core transcription factors and miRNAs maintain self-renewal in vitro and can even induce an ESC-like identity when ectopically expressed in somatic cells [63–66].

However, the pluripotent state in vivo is transient and in a state of constant flux, hence, although ESCs share many similarities with the early embryo, they also employ unique mechanisms to stabilize their state of pluripotency. Genes that regulate pluripotency in vitro are not necessarily required for early Epi development e.g. *Esrrb* and *Tbx3* [67, 68] and the cohorts of bivalent genes, those with both permissive (H3K4me3) and repressive (H3K27me3) epigenetic marks at their promoters, differ between embryos and ESCs [69–71]. Additionally, compared to the pre-implantation embryo, ESCs express high levels of repressive epigenetic factors [72], which may act to shut down the differentiation program. In fact, several thousands of genes alter their expression during ESC derivation [72] and, although the relevance of this is not fully understood, the majority function in growth and metabolism [72], suggesting that the current in vitro culture milieu may not accurately mirror the in vivo environment.

ESCs maintained in standard SL conditions are extremely heterogeneous (Figs. 2 and 3c). Global transcriptional analysis of single cells revealed two classes of heterogeneous gene expression, genes that are expressed bimodally, or those that are expressed in only a small number of cells, but at high levels – referred to as sporadic expression [73]. Subpopulations of cells have been identified that share transcriptional similarities, not only with the pre-implantation Epi [74–76] but also with endoderm [77] and later primed Epi [76, 78]. Furthermore, a subpopulation similar to the 2-cell embryo exists in ESC cultures, which exhibits expanded functional potency and can contribute to both embryonic and extraembryonic lineages in chimaeras [79–81]. *Zscan4*, specifically expressed within this population, is involved in maintaining telomere length, critical for ESC expansion in vitro [82–84]. It is currently unclear whether these subpopulations are also present in newly established ESC lines or if they emerge gradually in response to extended culture in the in vitro environment. As sampling of this 2-cell state maintains telomere length [82–84], it may arise as a mechanism to limit DNA damage during prolonged culture. Rather than marking stable factions of cells, the transcriptional heterogeneity among ESCs represents a dynamic landscape of interconverting states. In vivo, the blastocyst inner cell mass (ICM) is also a mix of Epi and primitive endoderm (PrE) precursors [85, 86] and, although there is no evidence that these populations interconvert during normal development [87], it is unknown whether this would occur if the period of pluripotency were to be prolonged, for example during diapause.

The source of ESC transcriptional heterogeneity has been variously attributed to cell cycle, transcriptional bursting, differences in colony size and density, partial differentiation or a combination of the above. As bivalent genes show a tendency towards heterogeneous expression, epigenetic modifications may also influence transcriptional dynamics [73, 88]. In reality, ESCs exist in a precarious balancing act of pluripotency and differentiation, prompted by their endogenous production of two factors with opposing functions: LIF, which supports self-renewal, and the differentiation-promoting factor FGF [89]. This signaling tug-of-war could conceivably manifest as cells that are transcriptionally teetering on the edge of different states. The fact that ESC cultures are further supplemented with exogenous LIF means that, although spontaneous differentiation occurs, in the majority of cases self-renewal wins. Consequently, blocking either signal forces cells in one or other direction. Withdrawal of LIF stimulates terminal differentiation while blocking FGF confines cells to a more homogeneous state of self-renewal, referred to as the “ground state” of naïve pluripotency [2, 90]. This ground state is achieved by culturing ESCs in defined serum-free medium (N2B27) with small molecule inhibitors of the MAPK/ERK pathway (PD0032, a MEK inhibitor) acting downstream of FGF and of Glycogen Synthase Kinase 3 (GSK3) (CHIR99021 or CHIR), together referred to as 2i medium [91] (Table 2). In this context, inhibition of GSK3 stimulates Wnt activity, which relieves TCF3-mediated repression of pluripotency-associated genes and prevents differentiation towards a later Epi state [92–94]. LIF is not required in 2i medium but enhances the efficiency of ESC clonal expansion [92]. ESCs cultured in 2i exhibit reduced expression of lineage markers and almost no spontaneous differentiation [95]. Blocking the processing of miRNAs in ESCs can stimulate a similar state of pluripotency [73]. However, single-cell approaches suggest that ESCs in these conditions are not entirely homogeneous as they contain rare cell populations similar to the 2-cell state and extraembryonic lineages [96, 97].

While SL and 2i are most commonly used to maintain ESCs, numerous other systems have been developed that support or enhance self-renewal including additional small molecule inhibitors [98–100], and synthetic serum substitutes, such as knockout serum replacement (KOSR) [101] (Table 2). While ESCs propagated in these distinct conditions all satisfy the functional definition of pluripotency, they have different morphological, transcriptional and epigenetic characteristics. When compared to embryos, SL-cultured ESCs are transcriptionally similar to E4.5–5.5 Epi and extraembryonic lineages [13, 46, 72], 2i–cultured ESCs correlate with earlier E3.5–4.5 ICM or Epi [13, 46, 97] (Fig. 1) and KOSR-cultured ESCs show a surprising transcriptional resemblance to endoderm [13, 46]. ESCs cultured in 2i and KOSR also display hypomethylated DNA relative to ESCs in SL [95, 102]. While these findings could be interpreted as different culture conditions capturing discrete points on the pluripotency spectrum, the majority of data have been acquired from bulk cultures and could instead represent changes in the relative levels of particular subpopulations. Single-cell transcriptomic analyses suggest that SL cultures are, in fact, comprised of two main cell populations, one similar to E2.5–3.5 embryos, and another corresponding to the Epi at approximately E5.5 [103]. It is therefore now understood to be the combination of these states that results in a bulk transcriptional signature similar to E4.5 embryos.

### (iii) functional potential

While transcriptional data provides some insight into the nature of ESC states, functional assays are required to assess pluripotential. When transferred to differentiation-promoting conditions in vitro or reintroduced into host embryos, ESCs can differentiate into derivatives of all germ layers. Traditionally, low-density monolayer differentiation was used as a simple, rapid means of testing functional potential. In serum-containing medium without LIF, ESCs form endoderm and mesoderm while neural differentiation requires serum-free medium (N2B27) [29, 104]. ESCs have also been reported to generate extraembryonic primitive endoderm (PrE) in vitro [105, 106]. However, as the majority of markers used to define these cells are also expressed in embryonic definitive endoderm, it is difficult to distinguish between these possibilities. A multitude of directed differentiation protocols have also been established, using cytokine and small molecule inhibitor cocktails to push cells more uniformly in a particular developmental direction. While the result is often a good approximation of the target cell type, on many occasions these differentiated cells are not fully functional. Therefore, a concerted effort is being made to develop 3D differentiation protocols, such as embryoid body (EB)-like structures or organoids, which more accurately mimic the complex in vivo environment [107, 108].

The gold standard test of pluripotency is whether cells can successfully incorporate into embryos and resume the normal developmental program. ESCs can incorporate into embryos at a range of pre-implantation stages [10, 46, 109–111], and contribute most efficiently to Epi-derivatives, but also at low levels to extraembryonic lineages [10, 46, 80, 97, 112]. The frequency of ESC contribution to extraembryonic cell types can be enhanced by selecting for particular subpopulations [77, 97] or by culture in 2i or KOSR [46, 97], consistent with the notion that ESCs maintained in these conditions correlate to earlier developmental stages than ESCs in SL and, as such, may have a less restricted functional potential. Remarkably, when reintroduced into developmentally compromised tetraploid embryos, live-born mice can be generated entirely from ESCs [113–116]. While the capacity of ESCs to generate primordial germ cell-like cells (PGCLCs) in vitro is limited [117], they can, albeit inefficiently, contribute to the germline in vivo following maturation through a later Epi state. In contrast, when ESCs are introduced into post-implantation embryos, they cannot integrate or differentiate [11] indicating that the naïve state of pluripotency is incompatible with the environment of the pluripotent post-implantation Epi.

## Epiblast stem cells

### (i) derivation and culture conditions

While ESCs resemble pre-implantation stages of development, PSCs have also been derived that are similar to the post-implantation Epi. These are referred to as epiblast stem cells (EpiSCs) (Fig. 1). ESCs and EpiSCs are distinct in behavior, morphology, growth factor requirements, transcriptional and epigenetic profiles and functional properties. EpiSCs can be derived from the post-implantation Epi from E5.5 until E8.0 [3, 17, 118], with decreasing efficiency at later embryonic stages [119] correlating with the gradual loss of pluripotency. Although the pre-implantation ICM rapidly differentiates in EpiSC medium [3], cell lines can be derived by first expanding the ICM in minimal medium then transferring outgrowths to conventional EpiSC conditions [120]. This primary step likely facilitates the developmental progression of the pre-implantation Epi to a state with later stage signaling requirements. Cells resembling EpiSCs can also be generated in vitro by long-term culture of ESCs in EpiSC conditions [16, 121] (Fig. 2). While, in vivo, Epi maturation from a naïve pre-implantation to primed post-implantation state occurs over approximately 2 days, in vitro this is a longer, selective process involving extensive cell death and differentiation [122]. Although ESC-derived EpiSCs (ESD-EpiSCs) are morphologically and transcriptionally similar to EpiSC lines derived from embryos, their full transcriptional profile or functional potential has not been directly compared.

EpiSCs are routinely maintained with FGF2 and ACTIVIN A (Activin) (FA conditions) [3, 17], a TGF-β family member with similar signaling properties to NODAL (Fig. 2, Table 2). In vivo, Nodal maintains the expression of the pluripotency markers *Oct4* and *Nanog* and prevents precocious differentiation of the Epi towards neural lineages [123, 124]. Similarly, in vitro, Activin regulates *Nanog* expression in EpiSCs [22, 125]. However, as *Nanog* ^*−/−*^ EpiSC lines can be generated, this is not the primary mechanism by which Activin signaling maintains self-renewal [118]. In EpiSC cultures, FGF, like Activin, blocks neural differentiation and may also prevent rare reversions of EpiSCs to an ESC-like state [3, 17, 22]. It is not clear whether FGF signaling plays a role in self-renewal of the post-implantation Epi in vivo, although it may regulate proliferation [126]. Whereas ESCs can be maintained in a relatively stable state of self-renewal, EpiSCs undergo high levels of spontaneous differentiation [127]. Dissociation of EpiSCs into single cells promotes cell death and differentiation, which can be reduced by using an inhibitor of Rho-associated, coiled-coil containing protein kinase (ROCK, Y-27632) [128] and passaging cells as clusters with gentle enzymatic dissociation or cell scraping. While ESCs are grown on gelatin, they produce endogenous fibronectin which is important for their self-renewal [129]. In contrast, EpiSCs in feeder-free conditions are grown on an exogenous source of fibronectin although there is limited evidence as to whether this influences the EpiSC state [130].

### (ii) transcriptional and epigenetic profiles

Consistent with in vivo development, EpiSCs show little to no expression of the naïve state markers *Rex1*, *Stella*, *Nr0b1*, *Gbx2*, *Klf4*, *Klf2* and *Fgf4* [3, 17] (Fig. 3). While expression of the core pluripotency-associated factors, *Oct4*, *Sox2* and *Nanog*, is maintained, *Oct4* expression is regulated mainly via its proximal enhancer element [59] and *Nanog* expression is reduced relative to ESCs [16, 131–133] corresponding to its downregulation upon embryo implantation [134, 135]. EpiSCs also express the early post-implantation Epi markers *Oct6*, *Fgf5*, *Otx2 Lefty* and *Nodal* [3, 17]. However, relative to their in vivo post-implantation Epi counterpart, EpiSCs exhibit elevated expression of markers of adhesion (e.g. *Tnc*, *Col1a1*, *Col6a1*), TGF-β, MAPK and Wnt-associated genes [119], all factors likely affected by the culture conditions. Compared to ESCs, EpiSCs express a distinct cohort of epigenetic regulators and miRNAs, exhibit an increasingly closed chromatin conformation and utilize distinct enhancer elements [133, 136–138]. The distribution of H3K4me1, a mark of enhancers and actively transcribed genes, varies significantly between ESCs and EpiSCs [138, 139]. This histone modification appears to play an active role in establishing the primed state of pluripotency as genetically perturbing the deposition of this mark results significantly enhances the spontaneous conversion of EpiSCs to a naïve ESC state [139]. EpiSCs also show reduced expression of SMARCAD1 relative to ESCs, a protein that is suggested to block H2K9me3-mediated heterochromatin formation [54].

As with ESCs, the fact that EpiSCs can be derived from a wide range of embryonic stages raises the question regards what in vivo stage, if any, they represent. The methylation status of specific promoters in EpiSCs is distinct from the in vivo post-implantation Epi [140] and, although EpiSCs were initially thought to correspond to the early post-implantation Epi around E5.0–6.0, they express markers of later, more differentiated cell types arising during gastrulation, e.g. primitive streak (PS) and mesoderm markers *Brachyury*, *Eomes*, *Gsc*, *Mixl1* and *Fgf8* as well as endoderm markers *Sox17*, *Gata6*, *Gata4* and *FoxA2* [3, 13, 17, 21, 119, 141, 142]. EpiSCs also express imprinted genes monoallelically [143] and female cell lines have an inactive X chromosome, observed in vivo from E6.5 onwards [16–18, 144]. Surprisingly, 2i–cultured ESCs exhibit a stronger correlation than EpiSCs to pre-gastrulation (E5.75) Epi [145] while EpiSCs are actually most transcriptionally similar to E7.25–8.0 embryos in which gastrulation is already underway [119].

In part these findings can be attributed to the extensive spontaneous differentiation of EpiSCs. This is supported by the fact that, in EpiSC cultures, endoderm genes are mostly expressed by differentiated, SSEA-1-negative cells [146]. However, subpopulations of cells exist that coexpress the lineage markers BRACHYURY and FOXA2 alongside the pluripotency markers OCT4, SOX2 and NANOG [142, 146]. Furthermore, the level of expression of markers of the mesoderm and endoderm is inversely correlated with the expression of neurectoderm markers such as *Sox1* [142], suggesting that there may be at least two subpopulations within EpiSC cultures. Isolated *Brachyury*-expressing cells show a propensity to differentiate [127], but they can expand and regenerate a mixed culture [142], indicating that this population, while unstable, is not on an irreversible path to differentiation. Interestingly, subpopulations of EpiSCs representative of later lineages, small fractions of EpiSCs also exhibit characteristics of naïve pluripotency. These cells regulate *Oct4* expression using its distal enhancer, express lower levels of *Brachyury* and *Fgf5* and high levels of naïve pluripotency markers [78, 131, 147]. Although this population is transcriptionally distinct from ESCs [131] it may represent an intermediate state between naïve and primed pluripotency. To add an additional layer of complexity, considerable variability exists between individual EpiSC lines. For example, some EpiSC lines express BRACHYURY in all cells, others have heterogeneous expression, while other lines do not express BRACHYURY at all. The relative composition of individual lines is also surprisingly stable as sub-clones maintained in different laboratories retain these characteristics [119]. The cause of variation between EpiSC lines is unknown and does not correlate with the stage from which these cell lines were derived or whether they are ESC or embryo-derived [119].

### (iii) functional potential

EpiSCs can generate derivatives of all germ layers both in vitro in cultured cells and in embryo grafting experiments. As with ESCs, when differentiated in vitro in serum-containing medium, EpiSCs mostly generate mesoderm and endoderm, while in serum-free medium they tend towards neurectoderm [17]. Removal or prolonged inhibition of FGF or Activin/Nodal signaling also stimulates neural differentiation [3, 17, 118]. While Wnt and BMP signaling maintain ESC self-renewal, these pathways stimulate EpiSC differentiation into a combination of mesoderm and endoderm lineages [127], consistent with their role during gastrulation (Fig. 2). It has also been suggested that BMP promotes extraembryonic endoderm and trophoblast differentiation [3]. However, as discussed above, many markers are shared between extraembryonic PrE and the epiblast-derived definitive endoderm, as well as between mesoderm and trophoblast; hence it is often difficult to distinguish between these fates using a handful of markers. Subpopulations of EpiSCs are biased towards particular lineages e.g. *Brachyury* positive cells demonstrate an enhanced capacity to form mesoderm [142] and, as EpiSC lines maintain distinct levels of *Brachyury*, they also vary in their differentiation efficiency towards particular germ layers [119]. The ability of BMP to induce EpiSC differentiation is via downstream activation of the Wnt pathway [127]. BMP and WNT3a also induce *Fgf8* and *Nodal* expression in EpiSCs [127], this is the same combination of factors that cooperate to initiate gastrulation in vivo, suggesting that differentiation in culture is recapitulating in vivo development.

Cells of the E6.0–7.0 Epi do not contribute to embryonic development when heterotopically introduced into blastocysts [148]. In keeping with this, EpiSCs also have little or no capacity to contribute to pre-implantation embryos [3, 17, 131, 149]. In rare cases of contribution, EpiSCs reactivate their X chromosome [149] suggesting that they have been reprogrammed to a naïve state. Although certain EpiSC subpopulations perform better in these assays [131] their contribution is still low. In part this could be due to adhesion-related incompatibility. ESCs, which can readily contribute to pre-implantation development, and EpiSCs are morphologically distinct; ESCs grow as compact, domed colonies while EpiSCs have a flattened morphology. Additionally, ESCs have homogenous and high levels of E-CADHERIN protein at cell-cell junctions, while EpiSCs have patchy, low levels at their interfaces [127]. Overexpression of *E-cadherin* (*Cdh1*) in EpiSCs in part rescues their contribution to pre-implantation embryos [149], but their contribution is still extremely limited, indicating that this is not the primary discordancy. Recently, EpiSCs were grafted into post-implantation host embryos, representing a closer stage-match regards their pluripotent state. While dissociated single cells could not incorporate, groups of cells efficiently integrated into the Epi, dispersing from the graft site and upregulated appropriate lineage markers [11]. EpiSCs incorporated most efficiently when introduced into mid or anterior PS, but remained as clumps when introduced into the posterior PS [119]. When *Brachyury*-positive EpiSCs were grafted into the PS of post-implantation embryos, they preferentially formed axial mesoderm and definitive endoderm [142] while *Brachyury* negative cells could not successfully incorporate into the embryo [11, 142]. This is consistent with the notion that *Brachyury* negative cells are comparable to the in vivo anterior neurectoderm which does not enter the PS. EpiSCs survive when grafted to later E8.5 embryos, when the host Epi is no longer pluripotent, but do not disperse or upregulate appropriate markers [11].

Although EpiSCs can contribute to all germ layers, they have not demonstrated germline transmission (GLT), mostly due to technical obstacles in assessing this capacity. In rare cases where chimaeras were obtained from EpiSC injection into pre-implantation embryos, GLT was not observed [3, 149], and it is not possible to re-introduce post-implantation chimaeras into recipient females for development to term. However, when grafted into post-implantation embryos, EpiSCs give rise to cells that express Alkaline Phosphatase, a characteristics of primordial germ cells (PGCs), in the region where germ cells arise [11, 142]. In vitro EpiSCs can generate PGCLCs only with low efficiency [145, 147] consistent with their transcriptional correlation to E7.2–8.0 embryos that have essentially lost their capacity to generate PGCs [150].

## In pursuit of intermediate pluripotent states

As discussed previously, multiple states of pluripotency have been captured in vitro including naïve ESCs, similar to the pre-implantation Epi, and primed EpiSCs, similar to the gastrulating post-implantation Epi. PSCs are a valuable model for studying and mimicking embryo development and a potentially useful tool for therapeutic purposes. However, to successfully differentiate PSCs into functional cell types in vitro, endogenous development will need to be recapitulated in a step-wise manner. Inevitably, in vitro differentiation is an imperfect imitation of in vivo development and therefore unnecessary ex vivo steps, for example the differentiation of naïve ESCs to a mature Epi state, might potentially introduce errors. Thus, it would be beneficial to access a stable intermediate pluripotent cell state, between ESCs and EpiSCs, equivalent to the post-implantation Epi prior to the onset of germ layer differentiation.

This distinct ‘formative’ state of pluripotency exists in the embryo at E5.5–6.25, when the naïve transcriptional program has been downregulated, but lineage-associated markers are not yet upregulated [151, 152]. Although there seems to be no clear consensus of what characteristics such a state would encompass, a number of conjectures can be made (Table 1). Whereas both the pre and post-implantation Epi express *Oct4*, the pre-implantation Epi employs the DE, while the post-implantation Epi the PE. In vivo this is not a binary switch [131], hence an intermediate pluripotent state may utilize both enhancers. Furthermore, cells of the early E5.0 Epi can contribute to embryonic development when introduced into blastocysts [148], hence we may expect cells in an intermediate state to maintain this capacity, but potentially also to contribute to development when introduced into post-implantation embryos. One clear-cut expectation is that an intermediate pluripotent state would efficiently generate PGCLCs in response to BMP. PGCs are induced in vivo in response to BMP signaling during a very defined time window. ESCs cannot efficiently produce PGCLCs, as BMP maintains naïve pluripotency, and conversely EpiSCs are representative of a developmental stage where PGC competence is already greatly reduced. Next we discuss a number of novel intermediate pluripotent states that have mostly been defined as exhibiting transcriptional profiles somewhere between naïve and primed states.

### (i) Epiblast-like cells (EpiLCs)

ESCs can differentiate to a state resembling EpiSCs by prolonged culture in FA (ESD-EpiSCs, see above). It is therefore tempting to speculate that this maturation recapitulates normal developmental progression bypassing intermediate pluripotent states that exist within the embryo. In support of this hypothesis, characterization of ESCs after 2 days in FA medium identified a transcriptional state similar to the E5.75 Epi [145], just prior to the onset of gastrulation. These culture conditions are almost identical to those for propagating EpiSCs with the addition of 1% KOSR to reduce cell death and induce a flattened morphology (Table 2). During these 2 first days, cells rapidly proliferate and show little cell death. Thereafter, on day 3, a wave of cell death is observed [145] corresponding to the initiation of the highly selective ESD-EpiSC program. Although cell death is observed in vivo at this time [153], it is not on a significant scale suggesting that, while the first 2 days of in vitro conversion to EpiSCs may represent a good model of this transition, later time points may not be representative. ESCs also transit through a state similar to the pre-gastrulation Epi after 2 days with FGF2 alone [154], although these cells have only been assessed using a limited set of markers and may have a compromised proliferative capacity [155] (Table 2). EpiLCs display hallmarks of pluripotency that are intermediate between ESC and EpiSC states; naïve markers, including *Stella*, *Rex1* and *Klf4* (Fig. 3) are downregulated while later differentiation-associated markers, such as *Brachyury*, *FoxA2*, *Sox17, Lefty*, *Sox1*, are not upregulated to the same extent as in EpiSCs and early Epi markers such as *Fgf5* and *Oct6* are expressed at the same or higher levels than in EpiSCs. *Nanog* is also transiently downregulated in EpiLCs [145] (Fig. 3), consistent with the fact that it is downregulated at peri-implantation stages and upregulated again in the PS of gastrulating embryos [134]. EpiLCs efficiently generate PGCLCs [145], but their full functional repertoire, including embryo contribution, has not been explored.

### (ii) a homogeneous ground state for primed pluripotency

Although EpiLCs represent a promising intermediate state between ESCs and EpiSCs, their transient nature means that any differentiation protocol still needs to begin with ESCs, and hence a stable intermediate pluripotent starting population would be highly desirable. ESC and EpiSC culture conditions have been variously adapted in pursuit of such a state. As discussed above, EpiSCs maintained with FA express later germ layer markers, and exhibit high levels of spontaneous differentiation. Eliminating this differentiation could push EpiSCs to a more homogeneous earlier Epi state. As with SL-cultured ESCs, endogenous differentiation-promoting factors destabilize the primed state of pluripotency. While FA mediates EpiSC self-renewal, it promotes differentiation when combined with BMP or Wnt signaling activation. EpiSCs express numerous WNT ligands [127], heterogeneously express *Axin*, a Wnt pathway component and show non-uniform β-CATENIN localization [142, 156], hence endogenous Wnt activity is a strong candidate for disrupting EpiSC self-renewal. In vivo, FGF, NODAL, BMP and WNT cooperate to initiate an epithelial-to-mesenchymal transition (EMT), and subsequent differentiation of cells in the proximal posterior region of the Epi [1]. Wnt signaling, downstream of BMP is required for mesoderm formation [157, 158]. Blocking Wnt signaling maintains the Epi in a prolonged state of pluripotency, while activation pushes the entire Epi to adopt a mesoderm fate [156]. Likewise, exposing FA-cultured EpiSCs to BMP4 or WNT3A recapitulates these events with cells undergoing an EMT followed by expression of mesoderm markers including *Brachyury*, *Nodal*, *Wnt3*, *Fgf8*, *Mesp1* and *Tbx6* [20, 127, 156].

Multiple reports have shown that disrupting Wnt activity in EpiSCs with small molecule inhibitors (XAV939, IWP-2, IWR-1) (Table 2) or genetic knockdown of *β-catenin* (*Ctnnb1*), promotes a more homogeneous primed state, enhances clonal expansion of single cells and derivation efficiency [20, 127, 142, 156, 159], and enriches for their capacity to undergo reversion towards ESCs [127]. However, when EpiSCs are derived in the presence of Wnt inhibitors, the resulting cell lines are, for as yet unknown reasons, dependent on these enhanced culture conditions. While EpiSC lines derived in standard FA can be reversibly exposed to the Wnt pathway inhibitor IWP-2 without negative effects, EpiSCs derived with FA and IWP-2 rapidly differentiate when IWP-2 is removed [146]. The long-term effects of Wnt signaling inhibition therefore need to be investigated in more detail. Wnt-inhibited EpiSCs (WiEpiSCs) have elevated expression of *E-cadherin,* and decreased *Snai1* and *N-cadherin* (*Cdh2*), potentially indicating a reduction in the fraction of cells undergoing EMT. Mesoderm and endoderm markers, such as *Eomes*, *Brachyury*, *FoxA2*, *Gata6*, *Sox17* and *Lefty2,* are downregulated and pluripotency markers are upregulated [127, 142, 146, 156, 159]. Global transcriptional analysis suggests that WiEpiSCs are similar to pre or early streak embryos [119, 127] or cells of the later Epi before entering the PS [159]. Therefore, blocking Wnt signaling inhibits differentiation, maintaining cells in a more robust state of self-renewal, akin to a developmentally advanced version of the naïve ESC “ground state” captured by FGF inhibition.

While naïve ESCs can generate chimaeras when injected into pre-implantation embryos, primed EpiSCs cannot. Although the functional potential of WiEpiSCs has been tested in chimaera assays, discrepancies in culture conditions (Table 2) and experimental design, between studies means that the data are difficult to interpret. WiEpiSCs maintained with FA and XAV939, or FGF and IWR-1, can contribute to all germ layers when grafted into post-implantation embryos [156, 159]. WiEpiSCs cultured with IWR-1 cannot contribute to pre-implantation embryos [159], but are cultured in the absence of Activin, which may limit their functional potential and the functional potential of XAV-treated cells has not been tested in pre-implantation embryos. WiEpiSCs cultured with IWP-2 have the capacity to contribute to embryonic development when injected into blastocysts [127], but their ability to contribute to post-implantation embryos has not been assessed. It therefore remains an open question as to whether these cells can contribute to both pre- and post-implantation stages of embryonic development.

Although Wnt signaling inhibition combined with FA maintains EpiSCs in a relatively stable primed pluripotent state, inhibition of Wnt signaling without FA leads to gradual differentiation [20, 156]. Intriguingly, a combination of small molecules that simultaneously inhibit (XAV939) and activate (CHIR99021 or IWR-1) Wnt activity, prevent differentiation in the absence of FA and can be used to derive EpiSCs directly from embryos [20], although it is not known whether these cultures are homogeneous (Table 2). The exact function of this cocktail is unknown, but involves sequestering β-CATENIN in the cytoplasm independent of its activity as a transcriptional regulator. Nevertheless, *β-catenin* mutant cells can be maintained in FA indicating that cytoplasmic β-CATENIN is not necessary for EpiSC self-renewal under normal conditions. While CHIR/XAV EpiSCs share many transcriptional hallmarks with FA EpiSCs, the naïve markers *Dppa2*, *Dppa4* and *Dppa5a* are more highly expressed and *Eomes* and *Nodal* are expressed at lower levels [20]. These cells may therefore exist in an intermediate state between EpiSCs and ESCs. CHIR/XAV EpiSCs can differentiate in vitro into all germ layers and form teratomas in vivo. However, unlike WiEpiSCs, they cannot generate chimaeras when injected into blastocysts, and their functional potential at later developmental stages has not been assessed [20].

In addition to the promiscuous expression of lineage markers in EpiSC cultures, there is also heterogeneous expression of earlier ESC and germ layer genes, marked by an *Oct4*-GFP reporter [131]. The effect of Wnt signaling inhibition on this population has not been assessed, however, EpiSCs uniformly expressing *Oct4*-GFP can be derived using FGF4 alone instead of FA [160] (Table 2). When the Epi of E5.5–6.5 embryos is explanted in FGF4 medium, most cells downregulate *Oct4*-GFP and likely differentiate but rare *Oct4*-GFP positive cells persist and can be purified by fluorescence activated cell sorting (FACS) [160]. Although FGF4-cultured EpiSCs (F4-EpiSCs) are pluripotent, they are distinct from *Oct4*-GFP cells in standard FA cultures, as they show reduced naïve marker expression and high levels of lineage markers. Therefore, while an apparently homogeneous, stable EpiSC culture can be maintained in FGF4 alone, it may represent a homogeneous late anterior PS-like state, similar to that described by Kojima et al. [119] rather than an intermediate. Heterogeneous expression of *Oct4*-GFP is reestablished when these cells are transferred to FA but it was not determined whether this was induced by Activin, distinct activities of FGF2 versus FGF4, or the disparity in FGF concentrations used (5 ng/ml FGF2 versus 25 ng/ml FGF4).

### (iii) combining naïve and primed culture conditions

Although Wnt signaling stimulates differentiation in the primed state of pluripotency, it stabilizes naïve pluripotency. It has therefore been suggested that culturing ESCs in a combination of primed culture conditions (FA), which normally coerce ESCs into EpiSCs, and Wnt signaling captures an intermediate state [155, 161]. ESCs cultured in FA and CHIR exhibit a mix of naïve and primed characteristics (Table 2) [155]. Although Wnt activity has previously been shown to induce *Brachyury* expression correlated with EpiSC instability in FA cultures [142], surprisingly this was not the case in FA and CHIR-cultured intermediate pluripotent stem cells (INTPSCs) [155]. INTPSCs retain ESC features including domed colony morphology, high clonogenicity, naïve marker expression (e.g. *Klf4*, *Rex1* and *Esrrb*), X chromosome activation and the capacity to contribute to embryonic development when injected into blastocysts [155]. They also acquire some EpiSC-like features such as the emergence of a subpopulation of cells coexpressing pluripotency (OCT4, ESRRB) and germ layer (FOXA2) markers. However, the majority of primed Epi markers, including *Fgf5*, *Wnt3* and *Otx2*, are expressed at levels intermediate between ESCs and EpiSCs. Although attempts were not made to derive INTPSCs directly from embryos, PSC lines have been derived from pre-implantation embryos under similar conditions with FA and a comparable Wnt pathway agonist, BIO with the addition of a LIF blocking antibody [161] (Table 2). While FGF, Activin, BIO stem cells (FAB-SCs) express intermediate levels *Fgf5*, associated with the primed state, and naïve miRNAs, they do not express other naïve markers at appreciable levels and are morphologically more similar to EpiSCs [161]. Furthermore, FAB-SCs are not functionally pluripotent as assessed by in vitro differentiation, teratoma assays and blastocyst injections [161]. While their capacity to generate teratomas could be rescued by LIF and BMP, it was not determined whether these cells had reverted to an ESC-like state.

Similar attempts were made to culture ESCs in the presence of the conventional ESC propagation component, serum (containing BMP activity), alongside the primed culture component Activin. Clonal assays selected for rare ESCs that can be maintained in an undifferentiated state in serum and Activin with similar efficiency to SL [162]. As forced expression of *Nanog* is sufficient to maintain self-renewal in the absence of LIF [74, 75] and *Nanog* is a direct target of Activin/Nodal signaling, this may explain how Activin maintains pluripotency in this context. These cells occupy a transcriptional midpoint between ESCs and EpiSCs expressing intermediate levels of naïve markers (*Rex1*, *Stella*, *Klf4*, *Nanog*) and primed/lineage markers (*Fgf5*, *Nodal*, *Lefty*, *FoxA2*, *Otx2*, *Gata6*, *Brachyury*), and are hence referred to as intermediate ESCs (IESCs) (Table 2). While not related to standard ESC or EpiSC culture conditions, a transcriptionally similar state can be generated by long-term culture of ESCs in conditioned medium (MEDII) from a human hepatocellular carcinoma cell line (HepG2) (Table 2). In contrast to IESCs which display a compact domed colony morphology [162], these early primitive ectoderm-like (EPL) cells have a flattened EpiSC-like morphology [163]. While EPL cells maintain the expression of some naïve markers including SSEA-1 and Alkaline Phosphatase, they downregulate others including *Rex1* and *Gbx2*. Furthermore, they upregulate the early post-implantation Epi marker, *Fgf5,* but not later PS or lineage-associated genes. Transferring IESCs or EPL cells to SL medium regenerates a standard ESC transcriptional profile indicating that these cells are not committed to their states. Neither IESCs nor EPL cells can contribute to embryonic development when introduced into pre-implantation embryos but their capacity to contribute to post-implantation development has not been assessed.

## Human pluripotent stem cells

As for mouse, multiple human states of pluripotency have been captured in vitro. Initial attempts to derive human ESCs (hESCs) by explanting the blastocyst ICM in mouse ESC (mESC) self-renewal conditions, SL, were unsuccessful [164, 165]. Instead, like EpiSCs, hESCs rely on FGF and Activin for self-renewal, exhibit a flattened colony morphology, limited clonogenicity, preferential use of the *Oct4* proximal enhancer and reduced expression of the naïve markers *REX1* and *TFCP2L1* compared to the human blastocyst ICM [3, 17, 125, 127, 166–171]. The X chromosome activation status varies between hESC lines, perhaps a result of inconsistency in the oxygen levels in different derivation protocols [166, 171, 172]. Furthermore, hESC cultures experience high levels of spontaneous differentiation and contain subpopulations that coexpress pluripotency and lineage markers including BRACHYURY and GATA4 [127, 173]. As with EpiSCs, Wnt pathway inhibition increases hESC homogeneity and reduces differentiation [127, 159]. Taken together, these findings indicate that the first human PSC state to be derived corresponds to a primed state of pluripotency. This begs the question as to whether nomenclature should now be re-aligned, and human ESCs be classified as human EpiSCs.

Initially it was not known whether a naïve human pluripotent state existed. One possibility being that this state is uniquely stabilized and more accessible in rodents due to their capacity to undergo diapause. Culturing hESCs in mESC ground state conditions (2i and LIF) results in extensive differentiation [174–177]. However, multiple modified small molecule inhibitor cocktails have now been identified that can induce naïve properties in hESCs [175–182], some of which also facilitate the derivation of cell lines directly from human embryos [175–177, 181]. Purported naïve hESCs share common phenotypic properties that differ from canonical primed hESCs including domed colony morphology, resistance to single cell dissociation, enhanced proliferation rate, increased X activation and decreased methylation at naïve loci [175–182]. Not surprisingly, the various, distinct combinations of inhibitors yield hESC states with vastly different transcriptional profiles. While several of these states transcriptionally cluster with naïve mESCs, rather than primed EpiSCs or hESCs [175–177], others are distinct from pluripotent states existing in the mouse. For example, Chan et al. [178] describe hESCs that upregulate the expression of genes associated with naïve pluripotency in mouse (*KLF2*, *KLF4*, *KLF5*, *DPPA3*, *DPPA5* and *NANOG*) but also significantly upregulate a long list of lineage-associated markers (*HNF4α*, *GATA6*, *GATA4*, *SOX17*, *FOXA2*, *T*, *EOMES*, *GSC*, *CDX2*, *WNT3*, *CDX2* and *NODAL*) and contain a fraction of cells that coexpress GATA6 and NANOG protein. Additionally, the majority of these cultures require FGF and Activin/Nodal signaling, and hence may be more similar to novel intermediate states of pluripotency described in mouse than bona fide naïve pluripotent cells. However, FGF and Activin signaling independence has been attained through genetic manipulation of hESC lines by forced continuous activation of STAT3 [183] or transient expression of *NANOG* and *KLF2* [184]. Notably, culture conditions comprising of the 2i inhibitors [91] alongside an inhibitor of protein kinase C (PKC) [184] can be used to successfully derive novel hESC lines in FGF and Activin independent conditions directly from embryos [181, 184].

Both naïve and primed hESCs generate derivatives of all embryonic germ layers when differentiated in vitro*,* or in vivo in teratoma assays [185–187]. Perhaps not surprisingly, naïve hESC states exhibit a reduced capacity to form mature cell types compared to primed hESCs when challenged with the same differentiation protocols. [188]. This is likely due to the fact that, for efficient differentiation, they must recapitulate the in vivo developmental trajectory and first transit to a post-implantation primed Epi state. While there are suggestions that hESCs generate trophoblast derivatives [127, 189–192], many of the genes used to define this cell type are also expressed in other lineages [193], leaving this issue unresolved. To determine whether hESCs maintain the capacity to contribute to embryonic development after periods in culture, they have also been introduced into mouse embryos. When primed hESCs are injected into mouse blastocysts, they persist at post-implantation stages, but negatively affect embryonic development [194]. Generally, hESCs also show poor contribution when grafted into post-implantation mouse embryos, but this can be improved by culturing hESCs in the presence of Wnt signaling inhibitors [159]. The capacity of naïve hESCs to contribute to pre-implantation mouse embryos is still unclear. While in some cases contribution has been observed, there is evidence that this is not reproducible and cells do not integrate [175, 176, 184, 195]. However, this is by no means an infallible experiment and potential inter-species incompatibility means that these results are difficult to interpret.

While hESCs share many properties with mouse EpiSCs, akin to the post-implantation Epi around gastrulation, they also show a strong correlation with EpiLCs, similar to the Epi at peri- or early post-implantation stages of development [196]. However, without access to post-implantation human embryos, there are limitations on temporally aligning human PSCs to in vivo development. Attempts have been made to correlate human states of pluripotency with mouse development, but transcriptional differences between species [197–199] suggest that this may not be the optimal approach. Nevertheless, single-cell RNA-sequencing of embryos from the more closely related cynomolgous monkey revealed that standard hESCs are indeed similar to the primate post-implantation Epi, while naïve hESCs show a stronger correlation with the primate pre-implantation Epi [196]. Recent advances in the ex vivo culture of human embryos allow development to implantation-like stages [200, 201], and therefore may also provide new insights into later human development. However, since there is no possibility for in vivo validation, conclusions should be drawn tentatively.

## Review and conclusions

While pluripotent cells are present in the embryo for a significant period of time during the course of development, for the past decade, in vitro PSCs have been limited to naïve ESCs and primed EpiSCs, perhaps suggesting that only a restricted number of stable attractor states can be isolated and stably maintained. However, recent developments in the field have revealed that these states can interconvert in vitro via distinct transient intermediates, hence the view of pluripotency as a continuum. Furthermore, modified culture conditions can induce novel characteristics in ESCs or EpiSCs confirming that the functional definition of pluripotency can be fulfilled while encompassing a broad spectrum of additional properties. What remains unclear is how these newly described states relate to one another, and whether bona fide counterparts exist in the embryo, neither of which are trivial questions to answer. Our current understanding is based on data generated using a range of basal media, cytokine combinations, cytokine concentrations, functional assays and transcriptional analysis platforms making direct comparisons near impossible. To understand whether the differences between these states are representative of an endogenous developmental progression, one needs to determine how each state functionally and transcriptionally relates to another in parallel controlled experiments, followed by comparisons to high-resolution data from the Epi population through successive stages of embryonic development. Ideally, these comparisons would be at a single-cell level, as heterogeneity within cell cultures, as well as in vivo within the Epi itself, may otherwise make these data challenging to interpret. The increase in the availability of single-cell data from both pre and post-implantation embryos suggests that this may soon be possible [202–206]. Furthermore, one must keep in mind that in vivo pluripotency is not a stable state, and there is no such thing as self-renewal, hence in vitro imitations will inevitably exhibit a certain degree of disparity.

## Abbreviations

2i: 2 inhibitor medium
DE: Distal enhancer
E: Embryonic day
EB: Embryoid body
EMT: Epithelial to mesenchymal transition
Epi: Epiblast
EpiLCs: Epiblast-like cells
EpiSCs: Epiblast stem cells
EPL: Early primitive ectoderm-like cells
ESCs: Embryonic stem cells
ESD-EpiSC: ESC-derived epiblast stem cells
F4-EpiSCs: FGF4-EpiSCs
FA: FGF2 and Activin A
FAB-SCs: FGF, Activin, BIO stem cells
GLT: Germline transmission
hESCs: Human embryonic stem cells
ICM: Inner cell mass
iESCs: Intermediate embryonic stem cells
INTPSCs: Intermediate pluripotent stem cells
KOSR: Knockout serum replacement
mESCs: Mouse embryonic stem cells
miRNAs: micro RNAs
PE: Proximal enhancer
PGCLCs: PGC-like cells
PGCs: Primordial germ cells
PrE: Primitive endoderm
PS: Primitive streak
PSC: Pluripotent stem cell
rESCs: Reprogrammed Epi ESC-like cells
SL: Serum and LIF
VE: Visceral endoderm
WiEpiSCs: Wnt inhibited EpiSCs
